# Bioactive Properties of Enzymatically Hydrolyzed Mulberry Leaf Proteins: Antioxidant and Anti-Inflammatory Effects

**DOI:** 10.3390/antiox14070805

**Published:** 2025-06-28

**Authors:** Yichen Zhou, Tianxu Liu, Rijun Zhang, Junyong Wang, Jing Zhang, Yucui Tong, Haosen Zhang, Zhenzhen Li, Dayong Si, Xubiao Wei

**Affiliations:** 1 Laboratory of Feed Biotechnology, State Key Laboratory of Animal Nutrition and Feeding, College of Animal Science and Technology, China Agricultural University, Beijing 100193, China; yczhou51@alu.cau.edu.cn (Y.Z.); wangjy9722@cau.edu.cn (J.W.); tracey9825@cau.edu.cn (Y.T.); s20223040758@cau.edu.cn (H.Z.); s20223040734@cau.edu.cn (Z.L.); dayong@cau.edu.cn (D.S.); 2 Beijing Key Laboratory for Animal Genetic Improvement, National Engineering Laboratory for Animal Breeding, Key Laboratory of Animal Genetics and Breeding of the Ministry of Agriculture, College of Animal Science and Technology, China Agricultural University, Beijing 100193, China; liutianx@cau.edu.cn

**Keywords:** mulberry leaf protein, enzymatic hydrolysis, oxidative stress, anti-inflammatory, natural antioxidants

## Abstract

Oxidative stress and inflammatory responses often occur concomitantly, and they are key causative factors in various human and animal diseases. Evidence suggests that mulberry leaf protein (MLP) may have potential antioxidant and anti-inflammatory properties, but there are significant challenges in enhancing their bioactivities. In this study, MLP was enzymatically hydrolyzed using papain, protamex, alkaline protease, trypsin, and neutral protease, followed by comprehensive evaluation of the antioxidant capacity, anti-inflammatory properties, and cytotoxicity of the hydrolysates. Our findings revealed that some enzymes significantly enhanced the peptide production and antioxidant activity of MLP (*p* < 0.01), and its activity was positively correlated with the degree of hydrolysis. Among the five hydrolysates, neutral protease hydrolysate (NeuH) exhibited the best antioxidant properties, with free radical scavenging rates of 71.58 ± 0.42% (ABTS), 26.38 ± 0.15% (OH), and 73.91 ± 0.37% (DPPH) at a concentration of 0.1 mg/mL. In addition, NeuH significantly suppressed IL-6 secretion (*p* < 0.01) and downregulated mRNA expression of IL-6, iNOS, and COX-2 inflammatory markers. This study not only establishes a correlation between enzymatic parameters and MLP biological functions but also demonstrates the potential of optimized MLP hydrolysates, particularly NeuH, as valuable natural antioxidant and anti-inflammatory ingredients for functional foods or nutraceuticals aimed at mitigating oxidative stress and inflammation-related disorders.

## 1. Introduction

Oxidative stress is characterized by the excessive accumulation of reactive oxygen species (ROS) that induce cellular damage through oxidative modifications of DNA, proteins, and lipid biomolecules [1]. Although ROS are important signaling molecules in the immune system, excessive accumulation of ROS activates many oxidative stress signaling pathways and in turn exacerbates the inflammatory cascades [2,3,4]. Current evidence indicates that pattern recognition receptors, including Toll-like receptors (TLRs), RIG-I-like receptors (RLRs), and NOD-like receptors (NLRs), recognize pathogen-associated molecular patterns (PAMPs) and damage-associated molecular patterns (DAMPs), which subsequently initiate mitochondrial- and NADPH oxidase-dependent ROS generation [5,6,7,8]. This redox signaling promotes the secretion of pro-inflammatory cytokines such as IL-1β and TNF-α/β, ultimately contributing to systemic tissue damage [9,10]. These findings collectively suggest a self-amplifying cycle between oxidative stress and inflammation in pathological processes [11].

Dietary antioxidant supplementation has emerged as a promising strategy for mitigating oxidative damage and improving health status [12,13]. In contrast, synthetic antioxidants like butylated hydroxyanisole (BHA) and butylated hydroxytoluene (BHT) have raised potential toxicity concerns [14,15]. Natural antioxidants are increasingly favored due to their superior safety and metabolic stability [16,17]. This paradigm shift highlights the importance of developing bioactive compounds from natural substrates. The antioxidant activity of proteins and peptides is fundamentally linked to their amphiphilic nature, which enables interactions with both hydrophilic radicals and hydrophobic cell membranes or oxidized lipids [18]. This dual solubility allows amphiphilic peptides to scavenge free radicals such as hydroxyl and peroxyl radicals at aqueous–lipid interfaces, chelate pro-oxidant metals, and disrupt lipid peroxidation chains [19]. Mulberry leaf (*Morus alba* L.), a traditional medicinal herb, has attracted considerable scientific interest as it contains a variety of bioactive substances including phenolic compounds, flavonoids, alkaloids, polysaccharides, and sterols [20,21]. Furthermore, it has been reported that mulberry leaf exhibits antioxidant and anti-inflammatory activities [22]. Mulberry leaf proteins exist predominantly as albumin, characterized by high β-sheet structures conferring exceptional thermal stability [23]. Their amphiphilic nature enables interactions with both hydrophilic radicals and hydrophobic membranes, facilitating antioxidant activities such as free radical scavenging and lipid peroxidation disruption [24]. Contemporary pharmacological studies have further validated the therapeutic potential of mulberry leaf proteins (MLP), particularly their antioxidant and medicinal properties [21,25,26,27].

However, the direct utilization of native MLP faces significant challenges. Native plant proteins often exhibit limited gastrointestinal absorption due to their high-molecular-weight tertiary structures and resistance to digestive enzymes [23]. Further, plant proteins may possess poor solubility, weak emulsifying capacity, and low thermal stability, restricting their application in functional foods or pharmaceuticals [28]. To overcome these limitations, enzymatic hydrolysis has become a critical strategy for converting native proteins into bioactive peptides. Proteases hydrolyze specific peptide bonds in proteins via cleavage site specificity, generating low-molecular-weight peptides [29]. These peptides exert bioactivities due to sequence-specific interactions with biological targets [30] Although, enzyme cleaving is sensitive to pH and inorganic salt, it has a lot of advantages such as specificity, safety, mild condition, and bioactive enhancement [31]. Thus, enzymatic hydrolysis has become the predominant methodology for bioactive peptide production, offering advantages in process safety, cost-effectiveness, and reaction controllability [32,33,34]. Previous investigations have demonstrated that protein hydrolysates from various sources, such as pepsin/trypsin-digested rapeseed protein and papain-hydrolyzed grass carp sarcoplasmic proteins, have significant antioxidant activity [35,36]. However, the antioxidant potential of MLP hydrolysates remains underexplored. Therefore, this study aims to optimize the parameters for the enzymatic hydrolysis of MLP into peptides, to systematically evaluate the anti-inflammatory and antioxidant activities of the hydrolysis products, and to elucidate the correlation between degree of hydrolysis (DH) and antioxidant activity.

## 2. Materials and Methods

### 2.1. Materials

Mulberry leaves (Guangdong Mulberry 69851) were purchased from the mulberry base of Long’an County, Guangxi Longhai Tianyuan Fruit and Vegetable Planting Family Farm. Mulberry leaves were air-dried, then oven-dried at 55 °C to constant weight. The dried leaves were pulverized in a crusher and sieved through a 60-mesh sieve to obtain mulberry leaf powder. MLP was isolated using the extraction procedures previously outlined by Chen et al. [37]. The powder was mixed with deionized water, and the pH was adjusted to 11.0. The mixture underwent ultrasonic treatment (235 W, 4.9 min) at 30 °C. After centrifugation (6000× *g*, 10 min), the supernatant was collected and adjusted to pH 4.0. Following 20 min incubation, the solution was re-centrifuged (8000× *g*, 10 min). The precipitate was washed twice with deionized water, redissolved, neutralized, and desalted for 48 h using a 3.5-kDa MWCO dialysis membrane. The extract rate was 34.12% and the desalted MLP extract was stored at −20 °C.

Penicillin–streptomycin mixture (100×), bovine serum albumin standard solution, BCA protein assay kit, SDS-PAGE gel preparation kit, papain (Pa), protamex (Pro), alkaline protease (Alk), trypsin (Try), and Coomassie Brilliant Blue G-250 were purchased from Beijing Solaibao Technology Co., Ltd. (Beijing, China). Neutral protease (Neu) was purchased from Aoboxing Biotechnology Co., Ltd. (Beijing, China). The CCK-8 assay kit was purchased from Beijing Lanjieke Technology Co., Ltd. (Beijing, China). N-1-naphthylethylenediamine hydrochloride and anhydrous p-aminobenzenesulfonic acid were purchased from Shanghai Macklin Biochemical Technology Co., Ltd. (Shanghai, China). ChamQ SYBR Color qPCR Master Mix (Without ROX) was purchased from Nanjing Vazyme Biotechnology Co., Ltd. (Nanjing, China). M5 Super qPCR RT kit with gDNA remover was purchased from Beijing Huagoumei Biotechnology Co., Ltd. (Beijing, China). IL-6 Elisa kit was purchased from Invitrogen Biotechnology Co., Ltd. (Shanghai, China). All reagents, unless otherwise specified, are of analytical reagent grade (AR).

### 2.2. Protease Activity and Enzymatic Hydrolysis of MLP

Protease activity was assayed following GB/T 23527-2009 [38]. Then, 0.2 mL of diluted enzyme was mixed with 0.2 mL of casein solution (20 g/L in 50 mM Tris-HCl, pH 7) and incubated at 70 °C for 10 min. The reaction was quenched with 0.4 mL of 65.4 g/L trichloroacetic acid (TCA). After vortexing and centrifugation (12,000× *g*, 4 °C, 5 min) (high-speed refrigerated centrifuge 5424R, Eppendorf AG, Hamburg, Germany), 0.4 mL of supernatant was mixed with 2 mL of 42.4 g/L Na_2_CO_3_ and 0.4 mL of Folin–Ciocalteu reagent (1 M). Following incubation at 40 °C for 20 min, absorbance was measured at 680 nm against a control (full-wavelength microplate reader SpectraMax M5E, Meigu Molecular Instruments, Inc., Rockville, MD, USA). A substrate-negative control was included. One unit (U/g) was defined as the enzyme amount, releasing 1 μg tyrosine per minute under assay conditions.

According to Monteiro et al. [39], MLP was dissolved in deionized water (1%, *w*/*v*) and heat in a water bath (DK-8A, Shanghai Precision Experimental Equipment Co., Ltd., Shanghai, China) at 90 °C to eliminate the interference of protease inhibitors in MLP. After cooling to room temperature, the mixture was hydrolyzed for 2 h under the conditions listed in Table 1. Then, the protease was inactivated by boiling in a water bath for 10 min. The mixture was centrifuged at 5000× *g* for 10 min, and the supernatant was collected. After dialysis (using deionized water) for 48 h using a dialysis bag with a molecular weight cutoff of 100 Da, the hydrolysate was freeze-dried to obtain the mulberry leaf protease hydrolysate, which was stored at −20 °C.

### 2.3. Degree of Hydrolysis

O-phthalaldehyde (OPA) was used to determine the DH according to the method of Nielsen et al. [40]. Samples were taken every 15 min. A volume of 0.2 mL of the sample was added to 1.5 mL of OPA reagent, mixed well, and left to stand for 2 min before immediately measuring the absorbance at 340 nm (OD340). The standard and blank groups used serine standard solution (0.9516 mM, 50 mg of serine was dissolved in 500 mL of deionized water) and deionized water, respectively. The DH was calculated using the following formula:

(1) $$Serine-{NH}_{2}=\frac{{OD}_{S}-{OD}_{B}}{{OD}_{St}-{OD}_{B}}\times 0.9516\;meqv/L\times \frac{0.1\times 100}{m}L/g\;protein$$

(2) $$h=\frac{Serine-{NH}_{2}-\beta }{\alpha }meqv/g\;protein$$

(3) $$DH(\%)=\frac{h_{1}{-h}_{0}}{h_{tot}}\times 100$$

where “Serine-NH_2_” represents the millimolar number of amino groups contained in serine in the sample; “OD_S_, OD_B_, and OD_St_” represent the OD_340_ values of the experimental group, blank group, and standard group, respectively; “α, β, and h_tot_” are taken as 1.0, 0.4, and 8.166 mmol/g, respectively [41]; “m” represents the mass of protein in the mulberry leaf protease hydrolysis product; “h_0_ and h_1_” represent the degree of hydrolysis relative to the original substrate before and after the hydrolysis reaction.

### 2.4. TCA-Soluble Peptide

Then, 3 mL of the sample was uniformly mixed with 3 mL of 10% (*w*/*v*) TCA solution. After standing for 0.5 h, the mixture was centrifuged at 5000× *g* for 10 min and the supernatant was collected. The protein concentration of the sample was determined before and after mixing using the Lowry method [42], and the values were recorded as T_B_ and T_A_, respectively. The yield of TCA-soluble peptide can be calculated using the following formula:

(4) $$\mathrm{Yield}\;\mathrm{of}\;\mathrm{TCA}\;-\mathrm{soluble}\;\mathrm{piptide}\;(\%)=\frac{T_{A}}{T_{B}}\times 100$$

where “T_A_, T_B_” represent the protein concentration of the sample before and after mixing.

### 2.5. Molecular Weight Distribution of Hydrolysate

Then, 160 μL of the sample (1.2 mg/mL) was mixed with 40 μL of loading buffer, and then the proteins were denatured by heating in a water bath at 100 °C for 10 min. After cooling to room temperature, the mixture was loaded into the wells of the protein gel. The method for preparing the protein gel is referred to in Table 2. The electrophoresis program was set to run at 80 V for 30 min, followed by 120 V for 60 min.

The molecular weight distribution was determined using a Waters 2695 high-performance liquid chromatography (HPLC) system equipped with a 2487 UV detector and workstation GPC software empower 3 (Waters Inc., Milford, MA, USA). A sample of 100 mg was dissolved in 10 mL of mobile phase (acetonitrile/water/trifluoroacetic acid, 40/60/0.1, *v*/*v*/*v*). After ultrasonication for 5 min, the solution was filtered through a 0.22 μm membrane filter, and 10 μL of the filtrate was injected into the HPLC system. The chromatographic column used was a TSKgel 2000 SWXL (300 mm × 7.8 mm), with a flow rate of 0.5 mL/min, column temperature of 30 °C, and detection wavelength of 220 nm. Cytoglobin (MW 12,384 Da), aprotinin (MW 6500 Da), bacitracin (MW 1422 Da), glycyl-glycyl-tyrosyl-arginine (MW 451 Da), and glycyl-glycyl-glycine (MW 189 Da) were used as standards for the molecular weight calibration curve.

### 2.6. Antioxidant Activity

#### 2.6.1. Reducing Power

The reducing power was tested according to Agnieszka et al. [43], where 1 mL sample (1 mg/mL) was mixed with 2.5 mL sodium carbonate buffer (0.2 M, pH 6.6) and 2.5 mL 1% (*w*/*v*) potassium ferricyanide, shaken vigorously, and incubated at 50 °C for 20 min. Then, 2.5 mL 10% (*w*/*v*) trichloroacetic acid (TCA) was added, and the mixture was centrifuged at 867× *g* for 10 min. The supernatant (2.5 mL) was combined with 2.5 mL deionized H_2_O and 0.5 mL 0.1% (*w*/*v*) FeCl_3_, left at RT for 10 min, and absorbance measured at 700 nm (OD_700_). Vitamin C (same conc. as sample) was the positive control.

#### 2.6.2. ABTS Radical Scavenging Activity

The referred method was from Chaturved et al. [44], with minor optimization, where 7 mM ABTS and 2.4 mM potassium persulfate solution (1:1) was incubated in the dark at RT for 16 h to form ABTS. This was diluted with PBS (pH 7.4) to OD_734_ = 0.70 ± 0.02. Then, 300 μL sample (0.1 mL/mL) was added to 2 mL ABTS working solution, incubated in the dark at RT for 10 min, and absorbance measured at 734 nm (A_1_). Vitamin C served as the positive control. The ABTS radical scavenging rate is calculated using the following formula:

(5) $$\mathrm{ABTS}\;\mathrm{free}\;\mathrm{radical}\;\mathrm{scavenging}\;\mathrm{ratio}\;\left(\%\right)=\left(1-\frac{A_{1}-A_{0}}{A}\right)\times 100$$

where A_0_ represents OD_734_ obtained by replacing ABTS with deionized water; A represents OD_734_ obtained by replacing sample with ionized water.

#### 2.6.3. OH Free Radical Scavenging Ratio

OH radical scavenging ratio was determined according to the method of Luo et al. [45], by adding 1.5 mL of salicylic acid (1.8 mM), 2 mL of ferrous sulfate (1.8 mM), and 1 mL of hydrogen peroxide (6 mM) successively to 1 mL of the sample (0.1 mL/mL). After thorough mixing by shaking, the mixture was allowed to react at 37 °C for 30 min. Then it was centrifuged at 3000 r/min for 5 min. The absorbance of the supernatant was measured at 510 nm (OD_510_) as A_1_.

(6) $$\mathrm{OH}\;\mathrm{free}\;\mathrm{redical}\;\mathrm{scavenging}\;\mathrm{ratio}\;(\%)=\left(1-\frac{A_{1}-A_{0}}{A}\right)\times 100$$

where A_0_ represents OD_510_ obtained by replacing salicylic acid with deionized water; A represents OD_510_ obtained by replacing sample with deionized water.

#### 2.6.4. DPPH Free Radical Scavenging Ratio

The procedure and methods of testing antioxidant activity referred to Zhou et al. [46], by adding 1 mL of the sample (0.1 mL/mL) to 2 mL of the DPPH working solution. After thorough mixing by shaking, the mixture was allowed to react in the dark at room temperature for 30 min. The absorbance was measured at 517 nm (OD_517_) as A_1_. Vitamin C was used at the same concentration as the sample and the positive control. The DPPH radical scavenging rate was calculated using the following formula:

(7) $$\mathrm{DPPH}\;\mathrm{free}\;\mathrm{redical}\;\mathrm{scavenging}\;\mathrm{ratio}\;(\%)=\left(1-\frac{A_{1}-A_{0}}{A}\right)\times 100$$

where A_0_ represents OD_517_ obtained by replacing DPPH with ethanol; A represents OD_517_ obtained by replacing sample with deionized water.

### 2.7. Cytotoxicity

According to the results of DH and test of antioxidant activity, NeuH showed good antioxidant capacity, so it was selected for the next step of the experiment. IPEC-J2 cells, HepG2 cells, and RAW 264.7 cells were diluted with complete culture medium (90% DMEM + 10% fetal bovine serum (FBS) + 1% penicillin–streptomycin (PS)) and then seeded into a 96-well plate and cultured for 24 h. For the experimental group, 100 μL NeuH samples diluted to different concentrations using DMEM were added into cells. For the control group, 100 μL DMEM was added into cells. For the blank group, only 100 μL DEME was added to the wells (without cells). After 24 h of cell culture, CCK-8 working solution (90% DMEM + 10% CCK-8) was added to each well. The plates were incubated until the OD_450_ of the blank group reached around 1.0. The cell viability was calculated using the following formula:

(8) $$\mathrm{Survival}\;\mathrm{rate}\;\mathrm{of}\;\mathrm{cell}\;(\%)=\frac{{OD}_{S}-{OD}_{B}}{{OD}_{C}-{OD}_{B}}\times 100$$

where OD_S_, OD_B_, and OD_C_ represent OD450 obtained as experimental group, blank group, and control group.

### 2.8. Anti-Inflammatory Activity

#### 2.8.1. Nitric Oxide (NO) Analysis

RAW 264.7 cells (5 × 10^4^ cells/mL) in complete medium (90% DMEM, 10% FBS, 1% P/S) were seeded (100 μL/well) in a 96-well plate and incubated overnight. Experimental wells received 100 μL serum-free DMEM with NeuH (100–800 μg/mL); blank and model groups received serum-free DMEM. After 6 h, LPS (100 ng/mL final conc.) was added to model and experimental groups, while blank received DMEM. After 24 h, supernatant NO levels were quantified via Griess reagent [47].

#### 2.8.2. Gene Transcription Level

RNA was extracted using Trizol (Beyotime Biotech, Shanghai, China), integrity checked by agarose gel electrophoresis, and conc. measured by microvolume spectrophotometry. RNA was reverse-transcribed to cDNA with the M5 Super qPCR RT kit (Mei5bio Biotech, Beijing, China). RT-PCR used ChamQ SYBR Color qPCR Master Mix (Vazyme Biotech, Nanjing, China) on a LightCycler^®^ 96 (Roche, Risch-Rotkreuz, Switzerland) to quantify inflammation-related gene expression (Table 3). Primers for GAPDH, COX-2, IL-6, and iNOS (Sangon Biotech, Shanghai, China) are listed in Table 4. qPCR conditions: 95 °C, 30 s; 40 cycles of 95 °C, 10 s and 60 °C, 10 s; melting curve: 95 °C, 5 s; 60 °C, 60 s; 95 °C, 1 s. mRNA levels were analyzed.

#### 2.8.3. Cytokine Analysis

After 24 h of LPS treatment, the cell culture supernatant was collected. The concentration of the inflammatory cytokine IL-6 in the supernatant was measured using an ELISA kit (Sigma-Aldrich, St. Louis, MO, USA).

### 2.9. Statistical Analysis

Each group of experiments was independently repeated three times, and the results were expressed as mean ± standard deviation. Excel 2019 and SPSS Statistics 27.0 were used for data processing and analysis. Graphpad prism 9.4.1 and Origin 2019 were used for diagram. One-way ANOVA was used for data analysis. *p* ≤ 0.05 was considered significant.

## 3. Results

### 3.1. Hydrolysis of MLP and Production of Peptides

**Figure 1:**
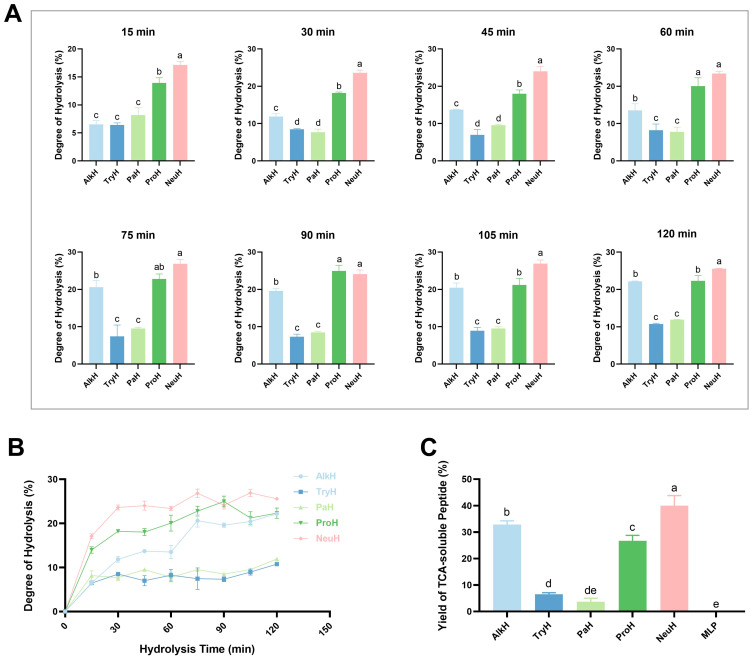
Degree of hydrolysis of mulberry leaf protein (MLP). (**A**) Degree of hydrolysis of MLP over time under different protease hydrolysis conditions; (**B**) hydrolysis degree curve within 2 h; (**C**) yields of TCA-soluble peptide ratio of MLP hydrolysate. Notes: AlkH, TryH, PaH, ProH, and NeuH represent alkaline protease, trypsin hydrolysate, papain hydrolysate, protamex hydrolysate, and neutral protease hydrolysate, respectively; different lowercase letters (a–e) indicate statistically significant differences between hydrolysates (*p* ≤ 0.05); same lowercase letters indicate no statistically significant differences (*p* > 0.05).

As shown in Figure 1A, the hydrolysis of MLP by different proteases varied greatly. The DH values of the five enzymes increased rapidly as the reaction progressed from 0 to 30 min. After 30 min, the rate of DH rise gradually slowed down for all these enzymes. TryH and PaH showed a similar tendency and reached their maximum DH around 90 min. The other hydrolysates reached their maximum DH at around 120 min. Figure 1B shows that NeuH had the highest DH value of 26.85 ± 1.17%, which was significantly higher than AlkH and ProH (*p* < 0.05), and significantly higher than TryH and PaH (*p* < 0.01). As shown in Figure 1C, the highest TCA-soluble peptide yield of 39.98 ± 3.82% was obtained from the neutral protease digest of MLP followed by AlkH and ProH, and the lowest yields of 6.55 ± 0.53% and 3.66 ± 1.37% were obtained for TryH and PaH, respectively.

### 3.2. SDS-PAGE and Molecular Weight Distribution

**Figure 2:**
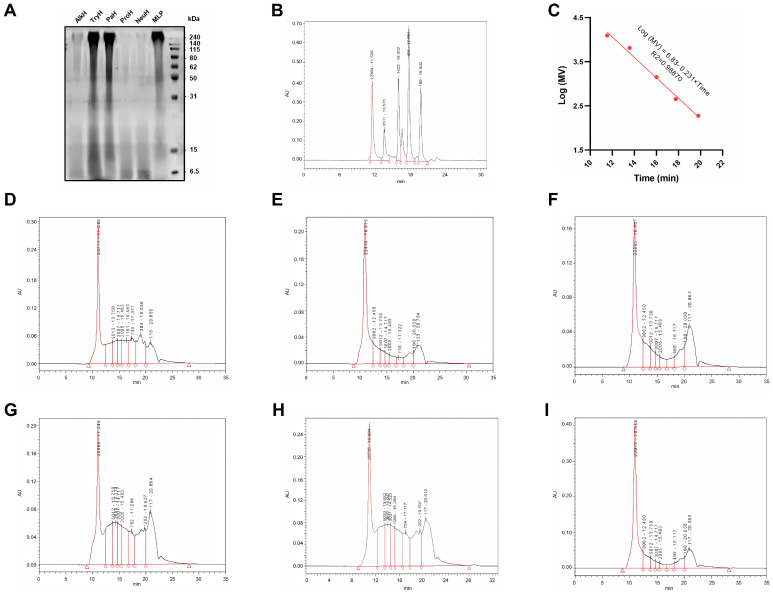
Molecular weight distribution of different hydrolysates. (**A**) SDS−PAGE electrophoresis of MLP and its hydrolysate; (**B**,**C**) scale curve for the determination of molecular weight distribution; (**D**–**I**) high performance gel permeation chromatogram of MLP, AlkH, TryH, PaH, ProH, and NeuH. Notes: AlkH, TryH, PaH, ProH, and NeuH represent alkaline protease, trypsin hydrolysate, papain hydrolysate, protamex hydrolysate, and neutral protease hydrolysate, respectively; MW means molecular weight; the “number-number” in (**B**,**D**–**I**) means “molecular weight (Da)-retention time (min)”.

The SDS-PAGE electrophoresis results of MLP and its enzymatic hydrolysates are shown in Figure 2A. Enzymatic hydrolysis resulted in an increase in protein components smaller than 31 kDa in the five enzymatic hydrolysates, particularly for AlkH, ProH, and NeuH. Correspondingly, there was a noticeable decrease in the protein components distributed between 50 and 240 kDa in the hydrolysates of these three enzymes compared with the pre-hydrolysis period. The calibration curve for molecular weight (MW) distribution is depicted in Figure 2B,C.

The high-performance gel permeation chromatograms of MLP and its enzymatic hydrolysates are shown in Figure 2D–I, and the molecular weight distribution is presented in Table 5. The results indicate that the protein components with MW greater than 10 kDa in MLP accounted for 56.24% of the total proteins. This was followed by TryH, PaH, AlkH, ProH, and NeuH. The protein components with molecular weights less than 1 kDa were most abundant in NeuH, reaching 41.50%. In the other four enzymatic hydrolysates, the proportion of protein components with molecular weights less than 3 kDa was significantly higher than that of MLP. DH of the five protease hydrolysates, from highest to lowest, was NeuH, ProH, PaH, AlkH, and TryH.

### 3.3. In Vitro Antioxidant Ability of MLP Hydrolysates

**Figure 3:**
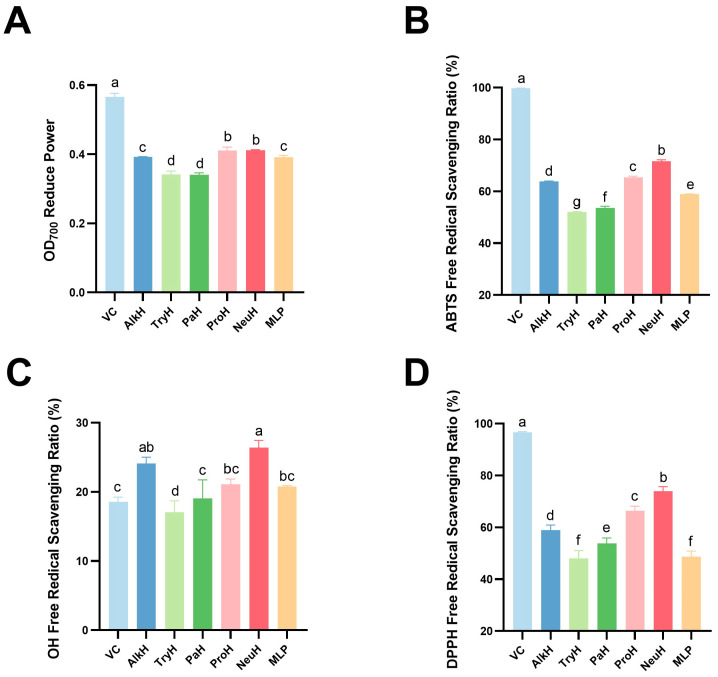
Antioxidant activity of different hydrolysates. (**A**) OD700 reduced power of different hydrolysates; (**B**) ABTS free radical scavenging ratio of different hydrolysates; (**C**) OH free radical scavenging ratio of different hydrolysates; (**D**) DPPH free radical scavenging ratio of different hydrolysates. Notes: VC, AlkH, TryH, PaH, ProH, and NeuH represent vitamin C alkaline protease, trypsin hydrolysate, papain hydrolysate, protamex hydrolysate, and neutral protease hydrolysate, respectively; different lowercase letters (a–g) indicate statistically significant differences between hydrolysates (*p* ≤ 0.05); same lowercase letters indicate no statistically significant differences (*p* > 0.05).

As shown in Figure 3A, NeuH and ProH had a greater reducing ability than MLP (*p* < 0.05), with 0.411 ± 0.002 and 0.392 ± 0.004, respectively. The OD_700_ values of the AlkH and MLP groups were similar (*p* > 0.05). However, PaH and TryH were significantly less antioxidant than MLP (*p* < 0.05). ABTS, OH, and DPPH free radical scavenging ratio of NeuH and AlkH were significantly higher than MLP (*p* < 0.05). For ABTS free radical (Figure 3B), ProH had a stronger antioxidant ability compared with MLP (*p* < 0.05), whereas PaH and TryH had a poorer antioxidant activity (*p* > 0.05). In the test of OH free radical scavenging ratio (Figure 3C), PaH and ProH had no significant differences compared with MLP (*p* > 0.05), but TryH group was significantly lower (*p* < 0.05). According to Figure 3D, all hydrolysates had a higher scavenging activity for DPPH free radicals than MLP (*p* < 0.05), except TryH, which had a similar scavenging activity as MLP (*p* > 0.05). Considering that NeuH is more capable in various aspects, NeuH was chosen to conduct further investigation. In addition, as shown in Table 6, the antioxidant activity of the enzymatic hydrolysis products of MLP was significantly (*p* < 0.01) correlated with the yield and degree of hydrolysis of TCA soluble peptides (*p* < 0.01).

### 3.4. Cell Cytotoxicity of NeuH

**Figure 4:**
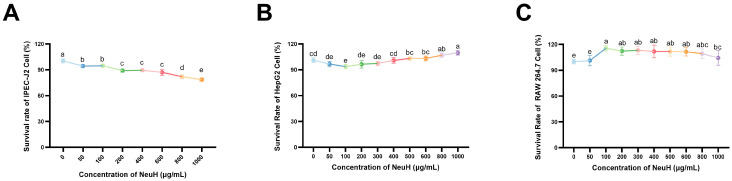
Effects of different concentration of NeuH on cell (*n* = 6). (**A**) Survival rate of IPEC-J2 cell; (**B**) survival rate of HepG2 cell; (**C**) survival rate of RAW264.7 cell. Notes: NeuH represents neutral protease hydrolysate; different lowercase letters (a–e) indicate statistically significant differences between different concentrations (*p* ≤ 0.05); same lowercase letters indicate no statistically significant differences (*p* > 0.05).

As shown in Figure 4A, the survival rate of IPEC-J2 cells decreased with increasing concentrations of NeuH. The survival rate of IPEC-J2 cells was 87.01 ± 3.41% at 600 μg/mL NeuH concentration. As the concentration of NeuH increased, HepG2 cell viability initially decreased and then increased (Figure 4B). The lowest survival rate of 93.69 ± 2.56% was observed at a concentration of 100 μg/mL. The cell survival rate was 106 ± 2.64% at a concentration of 800 μg/mL, which was significantly higher than that of the control group (*p* < 0.05). As demonstrated in Figure 4C, the survival rate of RAW 264.7 cells was significantly higher than that of the control group in the concentration range of 100 to 800 μg/mL (*p* < 0.05).

### 3.5. Effect of NeuH on LPS-Induced Inflammation in RAW 264.7 Cell

**Figure 5:**
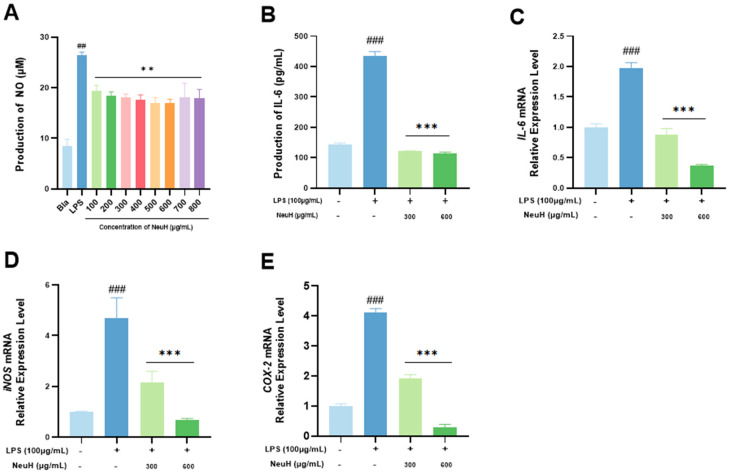
Effect on inflammation in RAW 264.7 cell of NeuH (*n* = 6). (**A**) Effect on NO production with NeuH in different concentration; (**B**) effect on IL-6 production with NeuH in different concentration. (**C**) Effect on IL-6 gene transcription with NeuH in different concentration; (**D**) effect on iNOS gene transcription with NeuH in different concentration; (**E**) effect on COX-2 gene transcription with NeuH in different concentration. Notes: Bla represents blank group; NeuH represents neutral protease hydrolysate; “##” means the difference is significantly compared with control group (*p* < 0.01), “###” means the difference is significantly compared with control group (*p* < 0.001), “**” means the difference is significantly compared with LPS group (*p* < 0.01), “***” means the difference is significantly compared with LPS group (*p* < 0.001).

Nitric oxide (NO) secretion was significantly higher in the LPS group than in the blank control group (*p* < 0.01), indicating that the inflammatory model was successfully established (Figure 5A). NeuH significantly reduced NO secretion of RAW 264.7 cells (*p* < 0.01), and a concentration-dependent trend was observed within the concentration range of 100–600 μg/mL. The effect of NeuH on the secretion of IL-6 cytokine by RAW 264.7 cells is depicted in Figure 5B. NeuH significantly reduced LPS-induced secretion of the inflammatory cytokine IL-6 (*p* < 0.01) in a concentration-dependent manner. LPS stimulation for 24 h significantly upregulated gene transcription of IL-6 (Figure 5C), iNOS (Figure 5D) and COX-2 (Figure 5E). Treatment with NeuH significantly downregulated the transcription of IL-6, iNOS, and COX-2 genes (*p* < 0.001) (Figure 5C–E).

## 4. Discussion

In recent years, oxidative stress and its associated pathologies have emerged as an active area of research [48,49,50]. The accumulation of oxidative stress contributes to the development of uremia and various vascular disorders [51,52,53]. Research has demonstrated that dietary supplementation with specific natural antioxidants can aid in the prevention of these conditions [54,55,56,57]. Consequently, the investigation of antioxidants is crucial for advancing human health. Numerous studies have reported that MLP enhances immune function through its antioxidant properties [58,59,60]. Studies indicate that protein hydrolysates exert beneficial health effects, including antioxidant, antihypertensive, and anticancer activities [61,62]. Therefore, in this study, we hydrolyzed MLP using five common proteases to enhance its bioactive properties.

Among the five enzymes tested, Neu exhibited the highest degree of hydrolysis (DH) for MLP, indicating significant variation in enzymatic efficacy toward the same substrate. This observation aligns with Bavaro et al. [63], who reported Neu’s superior activity and regioselectivity across diverse substrates—likely explaining its optimal catalytic efficiency in our study. Trichloroacetic acid (TCA) selectively denatures and precipitates high-molecular-weight proteins while exerting minimal effects on low-molecular-weight proteins and peptides [64,65,66]. Consistent with Neu’s high DH, NeuH yielded the highest TCA-soluble peptide content among the hydrolysates. Similar substrate-specific efficiency was documented for wheat germ (*Triticum vulgare*) globulin hydrolysis [67]. Given Neu’s status as the most prominent and extensively utilized enzyme [68], these findings hold broad relevance. Electrophoretic analysis revealed residual 50–240 kDa protein components in TryH and PaH, despite increased 6.5–15 kDa protein/peptide content. In contrast, AlkH, ProH, and NeuH showed near-complete hydrolysis of high-molecular-weight proteins into smaller fragments. Notably, substantial evidence confirms that low-molecular-weight proteins and peptides (<15 kDa) exhibit potent antioxidant and anti-inflammatory activities [69,70,71]. This strongly suggests that enzymatic hydrolysis potentiates MLP’s biofunctional properties through generation of bioactive peptides.

Previous studies have demonstrated that MLP possesses notable antioxidant properties [58,72]. This experiment further investigated the hydrolysis of MLP with proteases in vitro and found that the antioxidant activity of the hydrolyzed products surpassed that of MLP itself. Compared with MLP, hydrolysis with neutral protease at a concentration of 0.1 mg/mL increased the scavenging rates of ABTS, DPPH, and OH free radicals by 20.15%, 51.82%, and 26.90%, respectively, as well as the OD_700_ reducing power by 5.00%. Previous studies have indicated that the antioxidant properties of protein hydrolysates are closely related to their DH, with optimal hydrolysis conditions enhancing antioxidant activities [73,74]. However, not all studies align with this, e.g., excessive hydrolysis can disrupt the structure of active peptides, resulting in reduced activity [31,75]. Therefore, the relationship between DH and antioxidant activity is complex and may depend on the exposure sites of the active groups within the hydrolysates. As for hydrolysates of MLP, our results show that, with the higher DH, the bioactive peptides exhibited superior antioxidant capability, suggesting a potential positive correlation between DH and antioxidant activity.

Cell viability is a fundamental indicator of cell growth and can be used to evaluate the impact of culture conditions and the environment on cellular health [76,77]. In this study, HepG2, IPEC-J2, and RAW 264.7 cells, commonly used in antioxidant research, were selected for testing [78,79,80]. We found that, at a concentration of 100 μg/mL, HepG2 cell activity significantly decreased but, as the concentration of NeuH increased, cell activity gradually improved, even showing clear promotion of growth. The effect of NeuH on HepG2 cell viability was concentration-dependent, with a trend of increasing followed by decreasing activity, consistent with previous studies on the effect of renalase-derived peptides on HepG2 cell viability [81]. Surprisingly, the cells maintained good activity even with NeuH concentrations as high as 1000 μg/mL. Interestingly, IPEC-J2 cell viability decreased with increasing NeuH concentration, a result that appears inconsistent with similar studies [82]. A possible explanation is that IPEC-J2 cells may be highly sensitive to NeuH, leading to slight toxicity. For cell viability, 80–100% is generally considered to be non-toxic, 60–80% to be slightly toxic, and 40–60% to be moderately toxic [83,84,85]. In this experiment, the cell viability of IPEC-J2 at a NeuH concentration of 600 μg/mL was 87.01 ± 3.41%, indicating that NeuH is safe within the 0–600 μg/mL concentration range. Therefore, the effects of NeuH vary significantly between different cell types, with overall results suggesting that NeuH does not exhibit significant cytotoxicity and, to some extent, promotes cell growth.

Reactive oxygen species (ROS) not only induce oxidative stress but also mediate inflammation through the Toll-like receptor 4 (TLR4)/NF-κB signaling pathway [86,87]. ROS promote inflammatory cytokine secretion, activate downstream oxidant-producing enzymes, and generate additional ROS, thereby perpetuating a detrimental feedback loop that damages cells [88,89]. Antioxidant intervention can inhibit this cyclical progression [90,91,92]. In this study, we demonstrated that NeuH exhibits potent antioxidant activity. Consequently, we evaluated its potential anti-inflammatory effects using an LPS-induced RAW 264.7 cell inflammation model. LPS activates the NF-κB signaling pathway, leading to the upregulation of TNF-α, IFN-γ, IL-1β, IL-6, cyclooxygenase-2 (COX-2), and inducible nitric oxide synthase (iNOS) [93]. Consistent with previous reports [94,95], LPS stimulation significantly elevated the transcription levels of IL-6, COX-2, and iNOS genes, as well as IL-6 cytokine expression. Furthermore, LPS acts as an inflammatory inducer by stimulating macrophages to produce ROS via the TLR4 receptor [96]. Both the TLR4 receptor and its coreceptor MD2 are essential for macrophage recognition of LPS and play a pivotal role in LPS-induced inflammation [97]. NeuH treatment significantly suppressed these responses and reduced the expression of multiple inflammatory genes and cytokines. These findings suggest that NeuH exerts anti-inflammatory effects, at least partially, through inhibition of the TLR4/NF-κB signaling pathway. This mechanistic interpretation aligns with studies reporting that mung bean protein hydrolysate mitigates LPS-driven inflammatory cascades in macrophages via blockade of NF-κB nuclear translocation [98]. Similar anti-inflammatory activity has also been documented for soybean protein hydrolysates [99], thereby strengthening the foundational support for this study’s key conclusions.

In summary, we selected the optimal enzyme for degrading MLP from a group of common proteases and conducted a preliminary analysis of the relationship between DH and antioxidant activity. While our results suggest a correlation between these two factors, further research is needed to confirm and generalize these findings. Furthermore, this study did not conduct animal experiments to verify the efficacy in vivo. Overall, the results provide a theoretical basis for the development of NeuH as a food additive or medicine with both antioxidant and anti-inflammatory properties.

## 5. Conclusions

In this study, we found that in vitro enzymatic hydrolysis of MLP could enhance its antioxidant activity. The hydrolysates obtained from neutral protease exhibited the highest antioxidant and anti-inflammatory activities. Furthermore, these hydrolysates showed no significant cytotoxicity. MLP showed better antioxidant activity with higher DH, suggesting that there might be a potential correlation between the above two indexes. These findings provide theoretical and technical support for the utilization of mulberry leaf resources and the development of MLP as novel natural antioxidant and anti-inflammatory agents.

## Figures and Tables

**Table 1:** Optimum enzymatic conditions and actual enzyme activities of five proteases.

Enzyme	Labeled Enzyme Activity (10^5^ U/g)	Actual Enzyme Activity (10^5^ U/g)	Optimum pH	Optimum Temperature (°C)	Enzyme Addition (U/g)
Alk	20	14.85	10	45	6000
Try	25	4.79	8	37	6000
Pa	8	6.78	7	55	6000
Pro	1	0.91	7	55	6000
Neu	6	8.52	7	45	6000

Notes: Alk, Try, Pa, Pro, and Neu represent alkaline protease, trypsin, papain, protamex, and neutral protease, respectively.

**Table 2:** Protein gel configuration method.

Concentration of Gel	15%	5%
Volume	10 mL	5 mL
30%Acr/Bis (29:1)	5 mL	830 μL
1.5 M Tris-HCl (pH = 8.8)	2.5 mL	0
1.0 M Tris-HCl (pH = 6.8)	0	625 μL
10%SDS	100 μL	50 μL
10%APs	100 μL	75 μL
TEMED	10 μL	7.5 μL
H_2_O	2.3 mL	3.42 mL

**Table 3:** RT-PCR reaction system.

Component	Volume
2 × ChamQ SYBR Color qPCR Master Mix (without ROX)	10.0 μL
Upstream primer (10 μM)	0.4 μL
Downstream primer (10 μM)	0.4 μL
cDNA template	1.0 μL
ddH_2_O	8.2 μL

**Table 4:** RT-PCR primer sequence list.

Gene	Gene Accession Number	Sequence
*GAPDH*	AC166162	F: 5′-TGAAGGTCGGAGTCAACGG-3′
		R: 5′-TCCTGGAAGATGGTGATGGG-3′
*COX-2*	DQ874614	F: 5′-CTGCAAGTGCATCATCGTTGTTC-3′
		R: 5′-CTGCAAGTGCATCATCGTTGTTC-3′
*IL-6*	AC112933	F: 5′-TACCACTTCACAAGTCGGAGGC-3′
		R: 5′-CTGCAAGTGCATCATCGTTGTTC-3′
*iNOS*	AF427516	F: 5′-GAGACAGGGAAGTCTGAAGCAC-3′
		R: 5′-CCAGCAGTAGTTGCTCCTCTTC-3′

**Table 5:** Molecular weight distribution of MLP hydrolysate.

Sample	>10 kDa (%)	5–10 kDa (%)	3–5 kDa (%)	1–3 kDa (%)	<1 kDa (%)
MLP	56.24	7.83	3.92	4.68	27.43
AlkH	29.21	8.58	7.05	16.76	29.70
TryH	52.16	9.26	5.12	7.31	26.16
PaH	42.07	9.37	5.04	6.49	37.03
ProH	25.05	10.19	8.13	15.65	40.97
NeuH	23.66	10.71	8.31	15.92	41.50

Notes: AlkH, TryH, PaH, ProH, and NeuH represent alkaline protease, trypsin hydrolysate, papain hydrolysate, protamex hydrolysate, and neutral protease hydrolysate, respectively.

**Table 6:** Correlation analysis of the antioxidant activity of MLP hydrolysate with hydrolysis-related indexes.

Items	YTP	DH	RP	DPPH	ABTS	OH
YTP	1.000	0.931 **	0.917 **	0.873 **	0.868 **	0.959 **
DH	0.931 **	1.000	0.949 **	0.853 **	0.908 **	0.978 **
RP	0.917 **	0.949 **	1.000	0.000	0.920 **	0.983 **
DPPH	0.873 **	0.853 **	0.000	1.000	0.141	0.064
ABTS	0.868 **	0.908 **	0.920 **	0.141	1.000	0.964 **
OH	0.959 **	0.978 **	0.983 **	0.064	0.964 **	1.000

Notes: “YTP” represents “yield of TCA-soluble peptide”; “DH” represents “degree of hydrolysis”; “RP” represents reducing power”; “DPPH” represents “scavenging rates of DPPH”; “ABTS” represents “scavenging rates of ABTS”; “OH” represents “scavenging rates of OH”; “**” indicates highly significant differences (*p* < 0.01).

## Data Availability

Data will be made available on request.
